# Robotic training for medical students: feasibility of a pilot simulation curriculum

**DOI:** 10.1007/s11701-022-01508-4

**Published:** 2022-12-06

**Authors:** Anya L. Greenberg, Shareef M. Syed, Adnan Alseidi, Patricia S. O’Sullivan, Hueylan Chern

**Affiliations:** 1 Department of Surgery, University of California, San Francisco, CA USA; 2 Department of Medicine, University of California San Francisco, San Francisco, CA USA; 3 Department of Surgery, University of California, 550 16th Street, San Francisco, CA 94158 USA

**Keywords:** Undergraduate surgical education, Robotic surgery simulation, Feasibility of pilot robotic surgery training, Medical student education

## Abstract

While robotic procedures are growing rapidly, medical students have a limited role in robotic surgeries. Curricula are needed to enhance engagement. We examined feasibility of augmenting Intuitive Surgical (IS) robotic training for medical students. As a pilot, 18 senior students accepted an invitation to a simulation course with a daVinci robot trainer. Course teaching objectives included introducing robotic features, functionalities, and roles. A 1-h online module from the IS learning platform and a 4-h in-person session comprised the course. The in-person session included an overview of the robot by an IS trainer (1.5 h), skills practice at console (1.5 h), and a simulation exercise focused on the bedside assist role (1 h). Feasibility included assessing implementation and acceptability using a post-session survey and focus group (FG). Survey responses were compiled. FG transcripts were analyzed using inductive thematic analysis techniques. Fourteen students participated. Implementation was successful as interested students signed up and completed each of the course components. Regarding acceptability, students reported the training valuable and recommended it as preparation for robotic cases during core clerkships and sub-internships. In addition, FGs revealed 4 themes: (1) perceived expectations of students in the OR; (2) OR vs. outside-OR learning; (3) simulation of stress; and (4) opportunities to improve the simulation component. To increase preparation for the robotic OR and shift robotic training earlier in the surgical education continuum, educators should consider hands-on simulation for medical students. We demonstrate feasibility although logistics may limit scalability for large numbers of students.

## Introduction

Robotic surgery has grown over the last decade [1]. At present, its use spans multiple specialties and a wide range of common procedures that historically were performed using open or laparoscopic techniques [1]. To enable learner exposure to the field of surgery and acquisition of appropriate competencies across the surgical training continuum, surgical education must adapt to this shifting landscape.

Moreover, for medical students, surgical education has relied on active student engagement [2]. facilitated by direct proximity of the student to the surgical field, team, and instrumentation during open and laparoscopic cases. This allowed faculty and residents to offer students real-time teaching points and enabled students to have a role in the operation (e.g., retraction, suction, camera navigation). Robotic cases offer distinct advantages to students, such as the ability to visualize the operation three-dimensionally while sitting next to the operating surgeon at the robotic console and the ability to hear communication in the room through robotic microphones[3]. Other theoretical advantages may include ability to re-position themselves in the operating room (OR) to optimize their visibility of the monitor projecting the surgical view and opportunity to reference surgical resources on their mobile devices when not scrubbed in. However, the physical set-up of robotic cases is not conducive to the known benefits of the traditional paradigm. Whereas during open and laparoscopic cases surgical team members are huddled over the patient, in robotic cases everyone is physically distanced around the dark operating room (OR), separated by large centrally placed robotic equipment and the sounds it generates [3]. This predisposes students to disengagement and compromised learning given the inherent lack of orientation to the robotic equipment, inability to ask questions, perceived monotony of cases, and overall limited role in the operation [3].

As robotic surgery is an increasingly prominent part of core surgical clerkships (e.g., general surgery, obstetrics, and gynecology), strategies to enhance student engagement are needed. Educators have developed successful robotic curricula for surgical residents demonstrating significant performance improvement [4]. While curricula preparing medical students for robotic skills as residents are emerging [5], we lack curricula for active student role(s) during robotic cases, which is a failed opportunity to facilitate learning. This gap has particular implications for individuals bound for specialties that include robotics as optimizing preparedness for medical students may address the notably low robotic autonomy residents experience [6]. Specifically, introducing students to basic robotic skills may free residents up for more advanced robotic experiences.

Intuitive Surgical, Inc. (IS) offers introductory online training courses for OR staff, residents, fellows, and attending surgeons [7]. These courses complement IS-led in-person instruction focused on the robot technology and select role-specific content. This study examined the feasibility of augmenting an existing IS training in a pilot course for medical students. Specifically, our research questions were:

1. Could a curriculum adapted from the IS training be implemented with medical students in our setting?
2. Was the curriculum acceptable to the students?
3. What suggestions did students have to refine the pilot course?

## Materials and methods

Our institution’s surgical simulation center is loaned a da Vinci Xi trainer and simulation console for at least 2 weeks a year to support resident training. In June 2021, three half-days were reserved for medical student sessions allowing for this feasibility study. To maximize the hands-on nature of the course, we capped enrollment at six students per session, thus had capacity to accommodate 18 total learners. Our institutional review board approved the study as exempt; informed consent was obtained from all participants.

### Participant recruitment

Four weeks prior to the first session, medical students of all levels (including those completing a research year between third and fourth years) at our institution received a recruitment e-mail with information about the training and required pre-session 1-h online module. To indicate interest, students provided contact information, year in medical school, date availability, and attestation to their (1) understanding of the requirement to complete the module and (2) commitment to come to their assigned session. Students who participated in the course completed a pre-session questionnaire, which included basic demographic information, planned surgical specialty, and experience with robotic surgery. Students received no compensation for participation, nor did they have to pay to participate in the training.

### Structure of course

**Fig. 1:**
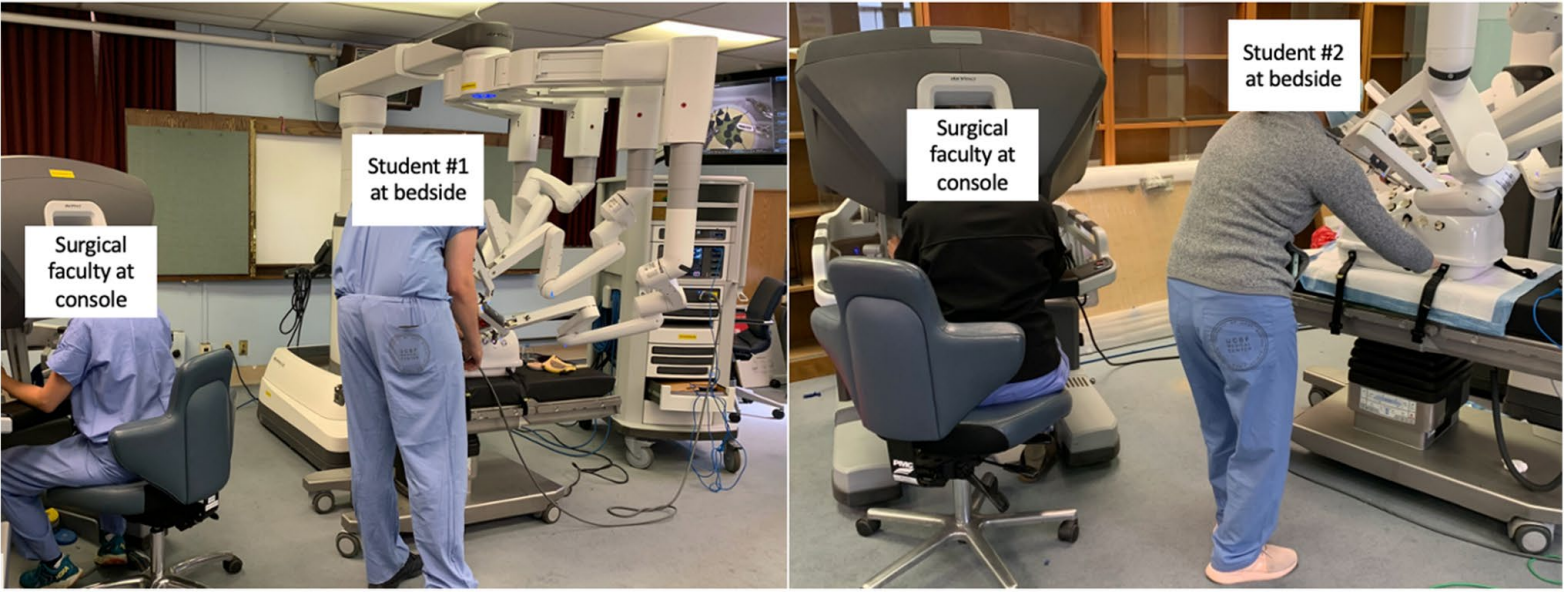
Participating students at bedside and surgical faculty at console of loaner robot during simulation component of robotic training

The course consisted of an online module and an in-person session. The course was tailored toward senior medical students and simulation of tasks performed by bedside assistants. Overall course objectives included introduction of students to robotic features, functionalities, and roles. Table 1 contains course details. The online module was selected given its introduction to components of the da Vinci Xi platform, terminology, and basic features. Within the in-person component, the robot overview and introduction to robotic console skills are part of the standard IS curriculum and were led by IS trainers. The simulation exercise, led by a surgical faculty, was added as a supplement to the standard IS curriculum (Fig. 1). Given the paucity of robotic curricula for medical students, the simulation was designed by our group. Specifically, the simulation consisted of introducing of a laparoscopic instrument through a laparoscopic port in a model abdomen while the robot is docked, bringing it into the field of view while navigating around obstacles within the model abdomen, and removing a suture handed off by the console surgeon. The obstacle within the model abdomen was created from a cardboard box covered in playdough; this enabled the instructor to see whether (and how deep) students hit the model as they were introducing the instrument into the field of view. This information was not used to assess student performance but rather to provide feedback to the instructor that the model was simulating what was expected. Students were also asked to exchange a robotic instrument and clean the robotic camera.

**Table 1:** Medical student robotic course details

Component	Purpose	Description	Total time	Approx. student time at robot
PART I: online module				
	To introduce students to robot fundamentals, including main components of the da Vinci Xi platform, terminology, and basic features	*“Essential multiport system fundamentals and da Vinci technical skills”* from existing the Intuitive Learning resource platform was selected At the end of the module, students completed an online assessment and received a certificate of completion, which they were asked to bring to the in-person session	1 h	–
PART II: In-Person Session (4 h)				
IS-led robot overview	To provide students a hands-on overview of the main robotic components	IS trainer reviewed standard terminology, pointed out features, and demonstrated functionality of the vision cart, the patient cart, and surgeon console Students had the opportunity to practice docking, undocking and repositioning the robotic arms and exchanging instruments Surgical faculty provided examples of when, by whom, and in what surgical context these functionalities would be employed in the OR	1.5 h	1 h
Introduction to robotic console skills	To give students an opportunity to experience the vantage point of the console surgeon	After a short demonstration by the IS-trainer, students took turns attempting ring transfer and suturing at the robot trainer console Simultaneously, other students completed various SimNow tasks at the simulation console with guidance from the IS trainer	1.5 h	30 min
Simulation exercise with surgical faculty*	To introduce students to the robotic bedside assist role	Students not actively participating in the simulation exercise were asked to wait in another room and each student was called to the robot individually To initiate the simulation, the console surgeon (surgical faculty) asked the student to introduce a laparoscopic instrument through a laparoscopic port in a model abdomen while the robot is docked Students were expected to communicate with the console surgeon while attempting to advance the instrument into the field of view, while navigating around obstacles placed within the model abdomen	1 h	10 min

*The simulation exercise was added as a supplement to the standard IS curriculum

Together, these tasks were intended to introduce students to several relevant competencies of the bedside assistant role, including introduction of instruments without injury of intraabdominal organs (safety), reaching a specific target within the abdominal cavity using two-dimensional view (visuospatial skill), using a laparoscopic instrument to perform specific task (instrument handling), exchanging robotic instruments (instrument exchange), and communicating with the console surgeon (communication). There was no formal assessment at the end of the simulation; students were allowed as much time as they needed to complete each of the simulation components.

### Determining feasibility

Feasibility was determined by assessing implementation and acceptability. For the implementation, we explored the extent this new training can be successfully delivered to intended participants. Implementation was assessed based on accessibility to a trainer robot, resources needed to carry-out the training, and student ability to sign-up and complete each course component.

For acceptability, we explored the extent the new training was judged as suitable, satisfying, or attractive to intended participants. Acceptability data came from the evaluation of post-session questionnaire responses and thematic analysis of three 30-min focus groups (FG; facilitated by non-surgeon surgical educator). The post-session questionnaire included three Likert-scale questions inquiring about value of the training, effect on preparedness for surgical sub-internship and effect on entry into surgical training. Students also answered three open-ended questions about the session: what was most valuable, what would make it more valuable, and what type of training should be offered for robotic surgery. Responses were analyzed using qualitative content analysis [8].

The FGs followed a semi-structured script exploring student perceptions of the session; FGs were audio-recorded, transcribed, and analyzed using inductive thematic analysis techniques [9]. One transcript was read by two authors and codes were identified using an inductive approach. The authors came to a consensus on the codebook and then applied the codebook to the other two transcripts. Codes were iteratively reviewed, discussed, and refined by the two authors. The coded transcripts were uploaded to Dedoose, a qualitative software [10]. Codes were reviewed to generate themes in the context of current aims. Team members noted their evolution of thinking about themes and considered their own reflexivity. One of the coders is an educator and the other is a medical student. Together they addressed potential biases from their respective perspectives as they generated themes.

## Results

### Participant Characteristics

While more than 600 medical students at our institution (including those pursuing concurrent degrees and completing a research year) received the recruitment email, 56 completed the initial survey to express interest in the robotic course. This included 18 third- and fourth-year students who had completed their core General Surgery rotation. Given our maximum course capacity of 18 students, we prioritized the third- and fourth-year students. We did not restrict enrollment to students who had prior experience in robotic cases or those with specific interest in specialties with emphasis on robotics.

Of the 18 students enrolled in the course, four students notified the training coordinators the week of the training that they would no longer be able to attend. Table 2 provides descriptive information about the 14 students (50% female, 86% in their fourth year) who completed the online-module and in-person training. All participating students had exposure to robotic surgery, with mean (SD) of 7.6 (7.9) cases. Notably, 8 (57%) participating students had previously served in the bedside assist role (either as an observer or a more active supervised participant) as part of their experience in the robotic OR.

**Table 2:** Characteristics of participating medical students

	No (%)
Gender	
Woman	7 (50)
Man	7 (50)
Not Listed	0 (0)
Year	
Third Year	2 (14)
Fourth Year	12 (86)
Specialty Consideration^a^	
General surgery	11 (79)
Urology	4 (29)
Obstetrics an Gynecology	2 (14)
Otolaryngology	1 (7)
Neurosurgery	1 (7)
Prior Roles during Clinical Robotic Cases^a^	
Observing	14 (100)
Performing Skin Closure	11 (79)
Serving as Bedside Assist	8 (57)

^a^Students were able to select more than 1 option

### Implementation

Though dependent on the infrastructure of and excess capacity from the existing resident robotic training, the loaner robot was accessible for the three medical student sessions. Table 3 outlines resources needed beyond the loaner robot and IS trainer. Students could sign-up for the course and complete each of the course components.

**Table 3:** Resources needed for course implementation

Role	Role description	Specific tasks	Time	Implied cost
Surgical faculty member	Provided leadership and oversight over training	Coordinated the trainer robot availability	1 h	$2,250 *(at $150/hour)*
		Designed the simulation exercise	1 h	
		Oversaw training preparation	1 h	
		Facilitated the training itself	12 h	
Medical student lead	Supported planning efforts	Issued recruitment survey	2 h	$510 *(at $30/hour)*
		Coordinated student communications	2 h	
		Assembled the simulation exercise obstacle	1 h	
		Supported the flow of the training itself	12 h	
Surgical skills lab operations manager	Supported logistical considerations	Reserved room for training	20 min	$40 *(at $40/hour)*
		Procured materials for simulation exercise obstacle	20 min	
		Provided snacks for training participants	20 min	
*Total:*			33 h	$2,800

### Acceptability

#### Post-session questionnaire

All participating students completed the post-session questionnaire. All found the training very or extremely valuable, thirteen (93%) felt much or extremely more prepared for their next surgical sub-internship, and all felt much or extremely more prepared to enter surgical training. Open-ended responses indicated that students appreciated the training’s hands-on nature, the ability to gain bedside assist experience, and the simulation exercise. Suggestions included shortening the IS-led robot overview favoring more simulation-based learning and allowing more time at the console to practice console skills such as knot tying. To augment robotic surgery training, students requested additional sessions, particularly immediately prior to the start of a surgical sub-internship.

#### Focus groups

Thirteen (93%) students participated in the three FGs. Comments reflected 4 themes: (1) perceived expectations of students in the OR; (2) OR vs. outside-OR learning; (3) simulation of stress; and (4) opportunities to improve the simulation component.

Regarding expectations of students in OR, students revealed their perception of the importance of having foundational robotic skills and being able to help with robotic cases.

“When I went to my first robotics case, I didn’t know anything. And even filling out the survey today, all the knowledge I got was from secondhand knowledge, when there was a break, from the intern [who is not in the case]. It was like, “Yeah, do this, this and this.” Maybe I watched a YouTube video or something, but it was mostly of the surgery itself, not about the setup. And **I know that as a sub-I it’s really important to learn how to do the setup and understand where the buttons are and how to manipulate stuff.** I thought it prepared me for that. So I at least, finally, got some good foundation.” (FG3-participant 1)

In fact, students perceive their performance ratings from residents and faculty to be dependent on this foundational knowledge.

“... **Faculty love it because they’re like, “Oh, she already knows. She can already help.” We get better reviews.** It’s not our fault whether somebody wants to take the time to teach us.” (FG3-participant 2)

Generally, they obtain knowledge piecemeal from disparate, unstructured sources and feel that an introductory robotic training addresses these gaps.

Regarding OR vs. outside-OR learning, students acknowledged that, without an adequate knowledge base, stepping in to help (or learn) is not appropriate in the OR because mistakes are high-stakes. Outside-OR robotic training with simulation, on the other hand, was felt by students to offer a safe place to learn and make mistakes.

“Now I feel confident being able to go in [to the OR] and just be like, “Okay, I have the training. I’m still learning, but I feel confident enough to help you.”… So I feel like this is still a safe environment to just learn. **You’re already concerned about the patient’s safety in the OR and everybody’s stressed out and trying to be efficient. So I feel that’s not the time to be worried about the medical student safety** versus here it’s safe enough to make your mistakes.” (FG3-participant 2)

Moreover, students reiterated that the current OR set-up, whereby a student is largely limited to watching the operation through the screen, is not conducive to learning about the robot. Students anticipate that a basic introduction to the robot outside of the OR will facilitate OR-based learning.

“**I always feel I get lost in the actual surgery watching the screen**, especially because it’s been mostly observation up to this point and just hearing people communicate in the background, but mostly focusing on anatomy and surgical steps and things that are going on the screen and in the body. **I think that now I’ll probably pay a little more attention to the other aspects of the surgery. The actual communication, the safety of the robot, and how everything is working outside of the patient**.” (FG3-participant 3)

Finally, outside-OR learning through training and simulation is critical due to the acknowledged lack of instruction in the OR.

“I can practice a heart exam on [someone]. I can practice almost everything else in medicine that’s not surgical outside of the OR. [For robot] there’s only these simulations, or nothing, or the OR. **So if you don’t have this, then it’s OR or nothing.**” (FG3-participant 1)

Regarding simulation of stress, students conveyed the value of the “uncomfortable” simulation exercise, which they felt facilitated learning not only directly through the stress it induced but also through creating a realistic (but low stakes) setting for practicing critical skills.

“[Learning something new] is inherently uncomfortable, but **that discomfort also cements a memory. And so I think personally, I wish there were more situations where we can be uncomfortable, but not have it be in a high pressure, high stakes environment**. So I wish there was more of this sort of training, personally. Because you don’t want to mess up when there's a real human in front of you.” (FG2-participant 2)

“I liked everything, and especially the last part [simulation]., because it was uncomfortable. . . **in this situation it was stressful, but it was nice to know that this is fake. And so now we can debrief what I was supposed to do.** And so, even though it was uncomfortable and stressful, and I'm pretty sure I would have completely obliterated some bowel, now I know how to not do that in the future.” (FG2-participant 1)

Finally, the theme regarding opportunities to improve the simulation component of the training focused on shortening IS led instruction, clarification and real-time guidance on performing the task.

“**I think it would've been helpful to have more specific direction** because it was like a little bit... I think this was probably simulated, but it was a little frenetic.” (FG2-participant 3)

One student expressed a lack of clarity around how the simulation connects with reality.

“I just think, for me, it's hard to wrap my mind [around simulation]. For me, as realistic as simulations can be, or as seriously as we should take simulations, **it's still a disconnect for me with reality.**” (FG1-participant 2)

## Discussion

This feasibility study of augmenting an existing IS robotic training for medical students supports three main findings. First, implementation of this pilot was successful. Second, the training was acceptable by the intended participants. Third, the value of a pilot was recognized. While robotic curricula for surgical residents have been developed [4], our findings contribute to the limited, yet growing literature base of medical student involvement in robotic simulation [5, 11–17]. However, much literature to-date has leveraged medical student naivety to the robot, laparoscopic surgery, and simulation to study various characteristics of the robotic learning curve [11–14] and, moreover, focused on the robotic console [11–16]. Few studies have aimed at developing robotic curricula specifically for medical students, with a particular emphasis on the bedside assist role where students may legitimately be engaged in the robotic OR, as we have [5].

The last decade has demonstrated successful implementation of numerous laparoscopic simulation courses for medical students [18–21]These courses have increased medical student knowledge and technical skill [21]; comfort and confidence in the OR [20]; and interest in surgery [19]. Though differently structured, these sessions included features similar to those in our pilot course, including pre-session work [20], hands-on instruction [18, 19], and simulation [19, 21], highlighting the precedent of such techniques in undergraduate surgical education and suggesting their potential role in robotic curricula for students.

We found that implementation of this pilot was successful. Students enrolled and completed each course component. The pilot required only modest resources. While access to the robot trainer itself was at no additional cost, course dates and spots available for students relied on its limited availability.

Expanding availability to all medical students would requires investment. In our current configuration, accessing the robot outside the semi-annual training events would necessitate blocking OR time, which carries implications of lost revenue and reduced patient access. However, a trainer dedicated to robotic simulation carries the price tag of a complete robotic system and, although in place at select surgical simulation centers [17], is cost prohibitive in many settings [24, 25]. More modestly priced alternatives, such as virtual reality or stand-alone console simulators, are increasingly adopted [26, 27]. However, these commercially available alternatives do not at present allow for simulation of the bedside assist role (i.e., robotic instrument exchange, introduction of laparoscopic instruments). Thus, incorporating robotic training as an element of medical student bootcamps (i.e., for those students who matched into a surgical specialty with emphasis on robotics) may be more feasible than holding such a training for all medical students.

Secondly, we identified through the students’ perception the value of this training, especially as preparation for future roles. Consistent with the literature [28, 29], students appreciated hands-on, clinically relevant training. While attending attitudes, interactions, and teaching; quality of feedback; and perception of self- improvement are most conducive to OR learning [30], these are limited in the robotic OR and students can feel intimidated, unwelcome, or ignored [31]. The physical separation from the surgical team and limited student role[3] puts students at risk for compromised learning and negative experiences in the robotic OR. Our study reinforced these sentiments and further revealed that students considered this training as an opportunity to increase their engagement and enhance their learning once they are in the OR during their sub-internship. Other studies also have highlighted the value of orientations prior to surgical experiences [32–34].

In the absence of structured robotic curricula or effective OR-based learning, students revealed that they are forced to self-identify learning resources or risk underperforming and causing patient harm in robotic cases. This is concerning as differences in patient outcomes with medical student participation are poorly understood [35]. Students further expressed that introductory robotic training with an “uncomfortable” simulation exercise created a low stakes but realistic environment that facilitated learning by “cementing the memory.” These perspectives align with ample literature demonstrating the value of simulation for a spectrum of learners [36]. While formal assessment of the efficacy of a robotic bedside assist curriculum is still needed, this type of opportunity may represent a pathway for students to serve as bedside assistants in robotic cases. In particular, while the present feasibility study included simulation of select, commonly-performed bedside assist tasks (e.g., introduction and advancement of laparoscopic instrument, robotic instrument exchange, camera cleaning), future iterations of the training may consider simulation of specific surgical scenarios (e.g., introduction of mesh and suture as part of a simulated robotic hernia repair) to offer students additional clinical context.

Third, this study found that students identified ways to improve the course without compromising feasibility. Students made three key suggestions. First, they identified a preference for shortening the IS-led robot overview in favor of more simulation-based learning. This reinforces the benefit of active forms of medical student learning (e.g., cases, simulation) compared to lecture-based learning [37, 38], though preferences have been mixed [38–40]. Zinski et al.[39] noted that as the students progress through medical training, their preference for simulation vs. lecture-based learning increases, a finding attributed to students’ increased tendency toward clinical application. Second, students expressed interest in spending more time at the console practicing skills such as knot tying. This sentiment may stem from desire for residency preparedness[41] or the perceived novelty of the technology itself [3]. Third, students recommended clarifying the steps of the simulation and enhancing real-time guidance. Educators advocate for these practices in higher education [42, 43] and OR-based learning [44], and they represent strategies that can be considered for future courses. We do not expect course adjustments to incorporate this feedback would negatively affect feasibility. If fact, they may actually enhance acceptability.

The simulation experience provides opportunity to explain the well-established connection between simulation and real-world performance [45, 46] to medical students. This is an important point as simulation-based learning is increasingly central to surgical education [47] and students can expect it during their future surgical training. Learner *buy-in* may be associated with engagement and performance [48].

Our study should be viewed in the context of several limitations. First, as this is a single-institution study, existing resources and incremental needs may vary when executing at other sites. For example, organizations with skills laboratories that have a robot trainer will not rely on IS loaners and may have more flexibility in timing and capacity of student sessions. As robotic surgery continues to grow and surgical simulation centers continue to expand, we may see concomitant growth of robot trainers at academic centers. Second, our feasibility study only included senior medical students who completed core surgical clerkships and were motivated to participate. Thus, their perspectives incorporate their experience of having previously participated in robotic cases, which may introduce bias. However, the training that focused on bedside assist tasks made this these students the appropriate audience. Future expansion of this training may include instruction of basic skills geared toward junior medical students and elicitation of their perspectives. Third, as a feasibility study, we only assessed medical student ability to sign-up and complete the training (implementation) and their perceptions of the training (acceptability). Content assessment to evaluate what students learned during the training was outside the scope of the current study and represents an important future direction to assess efficacy of the training. Fourth, acceptability of our training was based on perceptions of medical students; understanding the perspectives of other members of the robotic OR team is a critical next step in codifying curricula that prepares medical students to hold more active roles. In particular, a survey of surgical faculty perceptions of medical student involvement and engagement in robotic surgery is an important future direction for both developing effective curricula for medical students and ensuring faculty buy-in and awareness of this expanded education. Finally, effectiveness of the curriculum cannot be ascertained from the present study which addressed feasibility.

Despite these limitations, our study offers important insights into the feasibility of robotic training for medical students. As the field of robotic surgery continues to grow, structured and well-organized robotic training is becoming more prevalent [25, 26] and primarily reserved for post-graduate trainees. Given the barriers to medical student learning in the robotic OR [3], standard curricula preparing students to hold active roles are needed. Literature on robotic training specifically for medical students is limited at present and represents a gap our study begins to address.

## Conclusion

With the rise in robotic surgery, preparing medical students to hold active roles is important to enhance engagement and maximize learning. Hands-on robotic simulation training is feasible as preparation for meaningful robotic OR experiences, such as the bedside assist role. While our study revealed important suggestions to refine the course without compromising feasibility, feedback from other members of the robotic OR is needed to develop effective curricula, ensure buy-in, and increase awareness of this expanded education.

## Data Availability

The datasets generated and analyzed during the current study are available from the corresponding author on reasonable request.
